# Ticagrelor as an Alternative for Clopidogrel-Associated Acute Arthritis

**DOI:** 10.1155/2018/3786504

**Published:** 2018-04-08

**Authors:** Starr-Mar'ee C. Bedy, Jacob P. Kesterson, Greg Flaker

**Affiliations:** ^1^Pharmacy Department, University of Missouri Health System, One Hospital Drive DC060.00, Columbia, MO 65212, USA; ^2^Department of Emergency Medicine, University of Missouri, M562, One Hospital Drive DC029.1, Columbia, MO 65212, USA; ^3^Division of Cardiovascular Medicine, University of Missouri, One Hospital Drive, CE 306, Columbia, MO 65212, USA

## Abstract

A 65-year-old Caucasian man was hospitalized for a non-ST-elevation myocardial infarction. On discharge, the patient was started on multiple new medications, including clopidogrel and atorvastatin. Twenty-six days after discharge, he presented to the Emergency Department (ED) with polyarthralgias. He was instructed to stop atorvastatin and to follow up with rheumatology and cardiology clinic. At cardiology clinic follow-up 43 days after ED discharge, clopidogrel was discontinued and patient was switched to ticagrelor. On follow-up one month later, his symptoms had completely resolved. During the next 4 months, patient had routine follow-up due to participation in Cardiopulmonary Rehab and he had no cardiac events or recurrence of joint symptoms. Our patient had no history of arthritis. Because he initially presented with 2 medication classes associated with arthritis, each was withdrawn separately. The temporal association of patient's symptomatic improvement strongly suggests that the arthritis was caused by clopidogrel. Our patient was able to tolerate ticagrelor with complete resolution of his arthritis and no cardiac events. Clopidogrel-induced arthritis is a rare adverse drug event. For patients with a recent drug-eluting stent, alternative antiplatelet therapy with ticagrelor may provide positive cardiac outcomes without similar adverse effects.

## 1. Introduction

Antiplatelet medications that work by inhibiting the platelet P2Y_12_ receptor are increasingly used therapeutically: clopidogrel, prasugrel, ticagrelor, and cangrelor. Clopidogrel has been associated with a rare but severe adverse drug event (ADE) of polyarthritis in several case reports. Because of the significant therapeutic benefit associated with antiplatelet use in coronary artery disease, alternative treatment options are needed for patients who have an allergy or intolerance to clopidogrel.

We present a case of clopidogrel-associated acute arthritis which was treated on an outpatient basis. The arthritis resolved once clopidogrel was discontinued and ticagrelor was started. The patient had resolution of symptoms within 1 month of the treatment change.

## 2. Case Presentation

A 65-year-old Caucasian man had a recent hospital admission where he was diagnosed with an NSTEMI. At the time of admission, his past medical history was significant for hyperlipidemia and smoking and he had a family history of coronary artery disease. He received a stent to the LAD and PTCA to a diagonal vessel. During hospitalization, he experienced a cardiac arrest and developed a right bundle branch block requiring temporary pacing with subsequently placement of a permanent dual-chamber pacemaker. On discharge, the patient was started on multiple new medications including clopidogrel and atorvastatin.

Three and a half weeks after discharge, he presented to the Emergency Department (ED) with swelling, redness, and pain in his hands, feet, and elbow that began about one week prior. The symptoms began with swelling in his right index finger and this became painful and spread to the rest of his right hand over the preceding 3 days. On the day of presentation, he also noticed left anterior malleolar and midfoot pain that had been increasing throughout the day. The patient denied a history of gout, previous arthritic pain, or rheumatologic disorders. The patient's home medications were aspirin 81 mg daily, atorvastatin 80 mg daily, clopidogrel 75 mg daily, and metoprolol 25 mg PO BID and he reported compliance with his home regimen. He reported taking a statin about a year previously but could not confirm which statin or dosing and had not been taking any cholesterol medication prior to his hospitalization.

The patient reported subjective fevers and chills; however, on arrival he was afebrile and normotensive with a heart rate of 91 and pulse oximetry of 96% on room air. Physical exam findings were right hand swelling > left hand swelling; erythema overlying MCPs of right hand; decreased range of motion bilaterally due to swelling; sensation in tact; good capillary refill. Notable labs were WBC 17.64 × 10^9^/L, ESR 35 mm/hr, CRP 2.60 mg/dL. The patient's presentation was not consistent with an infectious etiology and he was discharged with watchful waiting, instructed to discontinue atorvastatin, and given follow-up with rheumatology.

He was seen by rheumatology a week later and there was concern for a new inflammatory arthritis triggered by the stress of his MI. His uric acid level was normal and he was started on prednisone. He initially responded well to prednisone but 2 days later his symptoms returned. He had a cardiology follow-up 2 weeks after ED presentation. Patient continued to experience the polyarthralgias at that time despite withdrawal of atorvastatin and addition of prednisone. Clopidogrel was discontinued and patient was switched to ticagrelor 60 mg bid. On follow-up one month later his symptoms of joint and muscle aches had completely resolved.

## 3. Discussion

A variety of medications are associated with arthritis and arthralgia and the mechanism is often through exacerbation of an underlying condition such as gout or rheumatoid arthritis. The PY12 inhibitor clopidogrel and HMG-CoA reductase inhibitors (aka statins) are associated with arthritis through an unknown mechanism [1–6]. These medications are often used together in patients with a history of stroke or coronary artery disease where there is strong evidence for decreased morbidity or mortality. Higher rates of statin-associated arthralgia/arthropathy have been reported with atorvastatin and simvastatin than other statins but it is unclear if this is due to prevalence of use or specific medication factors [1].

Our patient was younger with no history of arthritis. Because he initially presented with 2 medication classes associated with arthralgias, each was withdrawn separately. The temporal association of patient's symptomatic improvement strongly suggests that the arthragia was caused by clopidogrel. Our patient's presentation was similar to the other published case reports involving clopidogrel (Table 1). Additionally, we calculated the Naranjo Algorithm score for clopidogrel as 7 indicating a probable adverse drug reaction.

Clopidogrel polyarthralgias are a rare adverse drug event. For patients with a recent drug-eluting stent or other high risk conditions, alternative antiplatelet therapy should be considered. Two case reports evaluated use of prasugrel as an alternative antiplatelet agent and indicate that the arthralgia ADE may not be a thienopyridine class effect [5, 6]. However, prasugrel's black box warnings—such as use in patients with a history of TIA/stroke or weight < 60 kg—mean prasugrel may not be appropriate in all cases [7]. An alternative to prasugrel is needed in these patients and this is the first case reporting use of ticagrelor as an alternative.

Although ticagrelor postmarketing reports show a risk for joint pain/swelling, this is associated with gout and would have a different mechanism than clopidogrel-induced arthritis [8]. Our patient was able to tolerate ticagrelor with complete resolution of his polyarthralgias.

## Figures and Tables

**Table 1:** Comparison of patient presentation to published reports.

	Our patient	Case reports
Time of onset, days	14	10–21
Symptoms		
Fever	Subjectively present	May be present
Rash	None	Often present
Pruritus	None	May be present
Acute arthritis	Present	Present
Laboratory values		
WBC	Elevated	Elevated
ESR	Elevated	Elevated
CRP	Elevated	Elevated
Uric acid	Normal	Normal
